# Prevalence and Incidence of Multiple Sclerosis in Russian Federation: 30 Years of Studies

**DOI:** 10.3390/brainsci10050305

**Published:** 2020-05-18

**Authors:** Alexey Boyko, Mikhail Melnikov

**Affiliations:** 1 Department of Neurology, Neurosurgery and Medical Genetic, Prirogov Russian National Research Medical University, 1 Ostrovityanova st., Moscow 117997, Russia; boykoan13@gmail.com; 2 Department of Neuroimmunology, Federal Center of Brain and Neurotechnology, Federal Medical-Biological Agency of Russia, 1-10 Ostrovityanova st., Moscow 117997, Russia; 3 Scientific-Practical Center of Pediatric Psychoneurology, Michurinsky Prospekt 74, Moscow 119602, Russia; 4 Laboratory of Clinical Immunology, National Research Center Institute of Immunology, Federal Medical-Biological Agency of Russia, Kashirskoe shosse 24, Moscow 115478, Russia

**Keywords:** multiple sclerosis, epidemiology, prevalence, incidence

## Abstract

In the Russian Federation, multiple sclerosis prevalence rates vary from 10 to 80 cases per 100,000, depending on region and the nationality of the population. The main characteristics of multiple sclerosis epidemiology in the XX century in this big territory are: (1) steady increase in multiple sclerosis prevalence and incidence rates, maybe because of better diagnosis and treatment, but also changes in environmental/epigenetic risk profile and/or lifestyle factors; (2) increase of the female to male ratio, increase in multiple sclerosis incidence mainly in females; (3) appearance and increasing frequency of multiple sclerosis in ethnic groups, previously free of multiple sclerosis (Northern Tribes, Yakuts and others). The latest data show that in European Russia, the multiple sclerosis prevalence varies from 30 to 80 cases, in Siberia—from 20 to 70 cases, with steady increases, especially in women.

## 1. Introduction

Multiple sclerosis (MS) is characterized by uneven distribution worldwide, with increasing prevalence and incidence indexes in many countries [1]. The Russian Federation is located on area of 17,000,000 km^2^, where 191 ethnic groups live. Previous reviews of epidemiology of MS in Russia were published at the end of previous century, with some recent additions [2,3,4]. Although the methodology of epidemiological studies has improved significantly in recent years, many of them still do not provide sufficient data for analysis.

The majority of studies were published only in Russian. The objective for this review was to evaluate temporary changes in the prevalence of MS in various regions of the Russian Federation over a period of 30 years—from 1988 to 2018. Special attention is planned to be paid to the ratio of female to male (F/M) and ethnic origin of patients.

## 2. Materials and Methods

**Figure 1:**
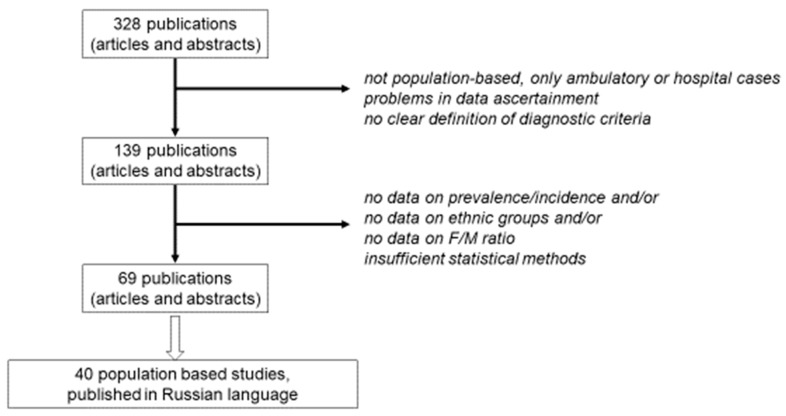
Methodology of the data selection.

According to the All-Russian National Bibliography, 328 articles on the clinical epidemiology of MS in the Russian Federation were published from 1988 up to now, including data collected from the late 1980s to 2018. All articles and abstracts were in Russian and mainly based on hospital cases, or had some problems in the calculation of widely used epidemiological indexes. The majority of studies used Poser criteria for MS diagnosis; only McDonald criteria of the last 10 years were dominating [5,6]. We selected the most qualified studies performed during this period, using these diagnostic criteria of MS, validated in Russia [7]. One hundred and thirty-nine publications were selected, some of which did not contain sufficient data. Finally, only 69 articles and theses with data from 40 studies (some of them in dynamics) were finally included in this review (Figure 1). We used only the average annual rates of prevalence and incidence rate among these populations at different time periods.

## 3. Results

### 3.1. European Russia

At the Northern Regions of the European part of Russia in the 1980s and 1990s, the prevalence of MS was near 30–40 cases per 100,000 population. In the Leningrad Region, the prevalence of MS was 28.8 cases per 100,000 population, with the incidence ratio varying from 0.9 in 1990 to 2.2 cases per 100,000 population in 1994 [8,9]. Later, in the Smolensk Region, the prevalence of MS at 2006–2008 was 47.4 cases per 100,000 population, reaching a maximum in the Glinkovsky district of the Region—up to 73.2 cases. For the F/M ratio, there was, on average, 1.6. For the period 2005–2010, the average annual prevalence of MS there reached 55.4 cases and the incidence rate—4.5 cases per 100,000 population with F/M ratio 1.6 [10,11]. The prevalence of MS in the Republic of Karelia for the period 2013–2018 was 61.2 cases per 100,000 population, the average annual incidence—2.7 cases per 100,000 population [12].

In the Central regions of European Russia, MS prevalence was 36 cases per 100,000 population in the *Nizhny Novgorod Region*, and 61 cases—in the Ryazan Region [13,14]. In the city of Nizhny Novgorod, the prevalence of MS in 2000–2002 was reported to be 38 cases per 100,000 population with F/M ratio of 1.7. The average annual incidence rate was 1.6 cases per 100,000 population [15]. In the Kaluga region in 2009–2012, MS prevalence was 54.7 cases per 100,000 population, with F/M ratio of 2.03 [16]. A longitudinal study in the Yaroslavl Region showed an increase in the prevalence rates from 42.6 cases per 100,000 population in 2004 to 56.2 cases per 100,000 population in 2015 and 78.5 cases in 2019. The incidence increased from 0.51 in 1975 to 1.58 in 2015 and 3.28 in 2019 [17,18,19,20].

In the Moscow Region in 2001–2005, the MS prevalence rates were 28.7–31.8 cases per 100,000 population, with a F/M ratio of 2.0 and an incidence rate of 2.1–2.6 cases per 100,000 population [21,22,23]. In Moscow, the first epidemiological population-based study conducted in 1989–1993 (Central District of Moscow, 120,000 inhabitants) showed the rate of MS prevalence—44.8 cases per 100,000 population with mean incidence rate—3.39 [3]. The next study, covering the period from 2008 to 2012, was carried out in another district of Moscow (North-Western District, 927,000 inhabitants). The data obtained were standardized for the European population. At this period the annual prevalence rate of MS was 55.6 per 100,000 population (the standardized rate—48.2) with a F/M ratio 2.6 and mean incidence rate—2.16 (the standardized index—1.88) [24,25].

In the South-Western Bryansk Region, the prevalence of MS increased from 40.6 cases in 2004–2008 to 48.1 cases per 100,000 population in 2012–2016. The average incidence rate in 2012–2016 was 1.9 cases, and F/M ratio—3.0 [26,27]. In the city of Orel, the prevalence of MS for the year 1995–2000 was 40.4 cases, incidence—1.45 cases per 100,000 population [28]. In the Kursk Region, the epidemiological study in 2015 showed a relatively high MS prevalence, with an average rate of 78.4 cases per 100,000 population (89.2 for towns and 63.2 for rural areas). The highest rates were in the towns of Sudzha (157.3) and Shigry (154.1), the lowest in Kurchatov (21.5) and Timskyi (26.7 cases per 100,000 population) rural districts [29]. In another study in the city of Kursk, the authors obtained similar indexes—the annual MS prevalence was 70.8 cases and incidence was 3.75 cases per 100,000 population [30].

Representatives of various ethnic groups of both Slavic and Turkic origin live in the Upper and Middle Volga region. MS prevalence rates there increased at the end of the last century. For example, in the Chuvash Republic, it was 19 cases in 1984–1990 and 31 cases per 100,000 population—in 1998–2002 [31,32]. A comparative analysis of the average annual indexes for one region of this Republic over 25 years of observation showed that MS prevalence increased from 16.7 cases in 1978, up to 37.6 cases in 2002 [33].

In the Republic of Tatarstan, the annual MS prevalence and incidence rates per 100,000 population was 32.0 and 3.3 in 1999, 34.0 and 5.0 in 2000, 36.7 and 5.5 in 2010. The dominating ethnic groups there are of Turkish origin. The prevalence rate in the capital of the Republic Kazan City was 34.9 cases per 100,000 population, with F/M ratio 2.4 [34]. The prevalence of MS at the neighboring Republic of Bashkiria (Bashkortostan) in 1999–2006 was 35.3 cases per 100,000 population (the standardized rate—31.9 cases per 100,000 population). MS was registered in the native ethnic group Bashkirs (Turks) 4 times less often than in the Tatars, and 2.5 times less often than in the Russians. MS incidence rate was 3.5 cases per 100,000 population [35]. A separate study conducted at the capital of the Republic, Ufa in 2010–2016 showed an increase in the prevalence of MS from 34.5 to 46.5 per 100,000 population [36]. In 2015, a local register of MS patients was created in this Republic. According to this register, the MS prevalence rate in 2008 was 31.3, the incidence rate was 1.0, and in 2018—47.9 and 3.0 cases per 100,000 population [37]. The authors proposed that the increase in MS frequency had been associated with both improved diagnostics plus the wide use of magnetic resonance imaging (MRI) and a true increase in MS incidence due to changing environmental factors.

The southern regions of European Russia traditionally have a great mix of ethnic groups with low MS frequency. In the Stavropol territory with a predominantly Russian population, the MS prevalence in 1986–1991 was 23.8 cases per 100,000 population. In the North-Western steppe areas of the region—32.8 cases, in the Central, Eastern and mountainous southern areas—22 cases [38]. The highest MS prevalence rate among the indigenous population at this time was observed in the small republics of the North Caucasus such as the Karachay-Cherkess Republic—16.9 cases [38]. In the neighboring Rostov region, also with a predominantly Russian population, in 2002–2006, the MS prevalence was 24.6 cases, and the incidence was 1.7 cases per 100,000 population. In the largest city of the region, Rostov-on-Don, the prevalence rate reached 26.3 cases per 100,000 population [39,40]. Later, another study in the same population showed that in 2016–2018, the MS prevalence in this city was 55 cases per 100,000 population, which was more than twice as high as in 2006. The authors also explained the detected increase in prevalence to both better diagnostics and increased activity of environmental factors [41,42,43]. The first epidemiological study in the Volgograd region in 1996–2000 showed that the MS annual prevalence was 31.9 cases, and the incidence was 9.8 per 100,000 population [44,45]. The highest rates were observed in ecologically unfavorable areas of the region—up to 52.4 cases per 100,000 population. The authors explained the higher incidence rate during this period by the optimization of medical care for MS with the organization of the MS center in Volgograd.

The mountainous territories of the North Caucasus are traditionally considered to be a low-risk area for MS development. The MS prevalence rate was 2–3 times higher in Russians born and living there than in the indigenous peoples of the Caucasus. Lower MS prevalence rates were found among representatives of the North Caucasian ethnic groups: the Chechens—13.7, the Circassians—8.0, Avars—14.6 cases per 100,000 population [46,47,48,49,50]. In the Republic of Dagestan, 2000–2004, MS prevalence rate was 5.7 per 100,000 population, and the incidence rate was 0.2 cases per 100,000 population, with a F/M ratio of 1.2 [47]. In the Republic of Ingushetia, the annual MS prevalence in 2010–2015 was 13.2 cases, and the incidence was 1.76 cases per 100,000 population. At the same time, the MS prevalence among the Russians living there was 45.0 cases, and among the Chechens—15.2 cases per 100,000 population. The average annual MS prevalence among the urban population was significantly higher (22.3) than among the rural population (6.3 cases per 100,000 population). The maximum prevalence was registered in the capital of the Republic, the city of Nazran—31.2 cases, and the minimum—in rural mountainous areas and the city of Karabulak—15.8 cases per 100,000 population [51,52]. In the Kabardino-Balkar Republic in 2006–2010, the prevalence in the capital city of Nalchik was 13.0, in 2010—13.7 cases per 100,000 population. In 2010, the MS prevalence among the Russians living in Nalchik was 34.3 cases per 100,000 population. At the same time, in rural areas of this Republic, the MS prevalence among the Russian population was 1.73 times lower than in the city of Nalchik (19.8 cases). The following study analyzing the MS epidemiology in the same population in 2012–2017 showed that the MS incidence rate was 1.9 cases per 100,000 population, with F/M ratio of 3.0 [49,50].

### 3.2. Ural Region

In the Ural region between Europe and Asia, MS prevalence rates at the beginning of this century ranged from 30 to 60 cases per 100,000 population. Later, in the Perm Territory in 2007–2011, the MS prevalence was 35.1 cases per 100,000 population, and the MS incidence increased from 3.2 in 1997 to 4.1 cases per 100,000 population in 2011 [53]. In the city of Chelyabinsk, located in the southern part of the Ural Mountains, the MS prevalence rate in 2005–2009 was 50.7 cases per 1,000,000 population [54].

### 3.3. Asian Russia

In Siberia, in 1994–1999, the MS incidence was highest in the largest city in the region *Novosibirsk* —2.6 cases per 100,000 population. Data from a 20-year prospective study of the population of this city showed an increase in annual prevalence rates per 100,000 population from 29.2 cases in 1994 to 54.4 in 2003, with incidence rates of 1.59 and 2.37 [55]. The first epidemiological study of MS in the *Tyumen region* showed that the MS prevalence rate in 2001–2006 was 24.8 cases per 100,000 population in the South area of this region, the highest level in the city of Tyumen—33.6 cases. The average MS incidence rate in this period was 2.5. The prevalence of MS in Northern Tribes was significantly lower in the Khanty ethnic group—5.8, in the Komi-Zyryansk group—16.2 cases per 100,000 population of this ethnic group [56,57,58]. The second analysis was carried out in the same population in 2014–2017, which revealed an increase in the MS prevalence up to 43.0 cases per 100,000 population. In 2017, the incidence rate increased to 4.0. The average F/M ratio was 2.4 [59,60]. The MS prevalence in the Khanty-Mansi Autonomous District in 2015–2018 reached 45.9 cases, the incidence—3.22, the F/M ratio—1.96. Among the indigenous population of the Khanty ethnic group, the MS prevalence in this period was still only 5.2 cases per 100,000 people of this ethnic group [61]. In the Tomsk Region in 2000–2004, the MS prevalence was 27.1 cases per 100,000 population, the standardized rate was 24.0 [62]. Later, in 2006–2010 in this region, the MS incidence was recorded at the level of 2.9 cases per 100,000 population. The F/M ratio increased from 1.5 to 2.0 [63].

Results of an epidemiological study of MS in the Altai Territory from 2010 to 2018 showed that MS prevalence rates increased from 41.2 to 56.3 per 100,000 population. There was 1.8 times higher MS prevalence in the cities of this region compared to the rural districts, F/M ratio was about 2.0. The incidence was 1.1 cases in 1998–2009 and 2.6—in 2010–2017. In some areas of this region, the prevalence of MS was relatively high, with more than 60 cases per 100,000 population—the town of Tselinny (75.4), the town of Mamontovsky (74.3 cases). In other areas, mainly rural, the MS prevalence was significantly lower; less than 10 cases per 100,000 population. The authors proposed the location of chemical and oil-processing enterprises in the territories with high rates, as well as the influence of environmental features of the urban environment, to be the possible reasons for this difference in the prevalence rates across the territories of the region with the same quality of medical care [64,65]. In the city of Irkutsk near the great lake Baikal in 2001–2005, the annual MS prevalence was 26.5 cases per 100,000 population, with F/M ratio 1.85 [66].

The first cases of MS in the native population of Siberia (the Yakuts) were identified in 1992–1995. The total MS prevalence there did not exceed 2 cases per 100,000 population; the majority of patients were Russian [67]. The first detailed analysis of MS epidemiology in the Sakha-Yakutia Republic was conducted in 2001–2006. MS prevalence was 22.5 per 100,000 population (standardized value was 21.2), and the MS incidence was 2.5 per 100,000 population (standardized value was 2.2). In the Russians, the MS prevalence was 36.1, incidence was 3.8, and in the Yakuts—13.4 and 1.8 for 100,000 population of this ethnicity, correspondingly. Average F/M ratio was 2.7, 2.4 in the Russians and significantly higher in the Yakuts—7.0 [68,69]. The second epidemiological study was carried out in 2008–2016. MS prevalence rate in this period increased to 30.3 cases per 100,000 population. The area with the highest risk of MS included the Oymyakonsky district (“Cold Pole”) with the MS prevalence 52.2 per 100,000 population. In the city of Yakutsk, the MS prevalence was 43.7 cases per 100,000 population. The authors noted a constant increase in the number of cases of MS, both among the Russians (prevalence 71.0 and incidence 2.8) and in the native population of the Yakuts—22.4 and 0.7 per 100,000 population [70]. The latest MS prevalence data in 2016–2018 showed the average MS prevalence there—34.2 per 100,000 population, reaching 57.3 among the European ethnic group (the Russians, the Ukrainians, the Germans etc.) [71]

In the Far East Region, the study of MS epidemiology in 1990s in the Amur Region with totally migrated population showed that the highest rates were in the Southern and some Central areas of this region, where the MS prevalence exceeded 50, and the MS incidence was more than 3 cases per 100,000 population. In Blagoveshchensk, the MS prevalence was 54.3 and incidence was 3.87 cases, in the Mikhailovsky district—58.3 and 4.26 per 100,000 population, correspondingly [72]. In the Northern mountainous districts, these ratios significantly decreased by up to 6.31 and 0.78 in the Tynda district. Notably, 80% of MS patients living in this area were from the 3rd and 4th generations of immigrants, i.e., their parents were indigenous residents of these places. At the same time in the city of Khabarovsk, the MS prevalence reached 40.5 with incidence—2.9 cases per year [73,74]. The study of MS epidemiology in the Far East regions in 2005–2010 showed the MS incidence in 3.95 cases per 100,000 population. In the Sakhalin Island, the F/M was 1.35 and in the Kamchatka Half-Island, it was 2.6. The underestimation of MS cases in the Northern area of the Far East with very low density of population in early 2000s could be explained by worse diagnoses, which was improved after organization of MS Centers with MRI after 2010 [75]. The latest published data showed the uneven frequencies of MS in this territory. MS prevalence and incidence rates were 23.4 and 1.1 per 1,000,000 population in the Primorye Territory, 33.0 and 4.15—in the Sakhalin Island, 30.1 and 3.48—in the Khabarovsk Territory, 31.4 and 1.54—in the Amur Region [76,76].

This section is divided by subheadings. It should provide a concise and precise description of the experimental results, their interpretation as well as the experimental conclusions that can be drawn.

## 4. Discussion

The main characteristics of MS epidemiology in various regions of the Russian Federation are:

– steady increase in MS prevalence and incidence rate, maybe due to better diagnosis and treatment, as well as changes in environmental/epigenetic risk profile and/or lifestyle factors;

– increase of the F/M ratio everywhere. The observed rise in F/M ratio cannot be easily explained by genetics or by new diagnostic technologies or increased awareness, which would apply to both sexes. Results of numerous population-based studies in different parts of the world and the meta-analyses suggest that the recent increase of MS incidence provide some evidence that this has primarily resulted from an increase in the incidence of MS among women [77,78,79,80,81,82];

– MS is registered now in all ethnic groups, though among Asian and Northern tribes, previously free of MS, the MS prevalence ratios are rapidly increasing. This was also seen within native Africans, the Maori population in New Zealand, and others ethnic groups [83,84].

Some possible factors that could contribute to this change are epigenetic/environmental risk/lifestyle factors or endocrine changes. The rise in the incidence and prevalence of MS in the world in the past decades paralleled the rapid socioeconomic development, urbanization, and westernization, which was marked by radical change in dietary and lifestyle habits. The industrial revolution and the contemporary age in Western countries gave rise to the fast-food industry and the widespread consumption of excessive salt, refined vegetable oils, and sugars and also led to reduced physical activity, exposure to artificial light at atypical biological times, and insufficient and poor-quality sleep [85,86,87,88]. The influence of other environmental factors, such as Epstein–Barr infection, vitamin D levels, smoking, obesity, and geographical location, changes in micro (in families) and macro levels (on population level) might cause epigenetic modification of predisposition. Vitamin D deficit due to spending less time outside and closed clothes, smoking and changes in diet, as well as the wide use of antibiotics, and the modulating microbiome could influence microRNA levels, DNA methylation and histone modification [89,90]. These epigenetic mechanisms are different and could depend on different factors [90].

**Figure 2:**
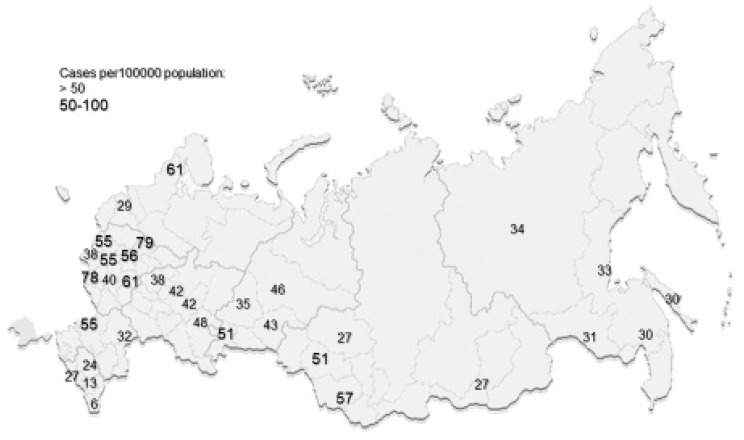
The latest data on MS prevalence per 100,000 population in regions of the Russian Federation.

**Figure 3:**
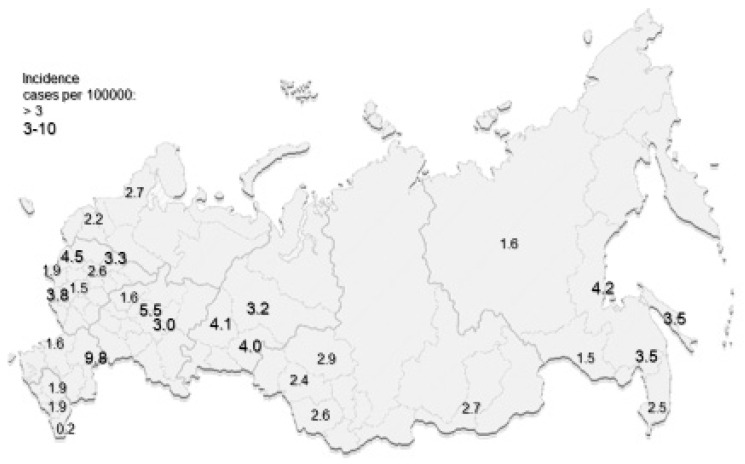
The latest data on MS incidence per 100,000 population in regions of the Russian Federation.

Results of the main and latest studies are presented in Table 1 and on the map of the Russian Federation (Figure 2 and Figure 3).

## Figures and Tables

**Table 1:** Data from the recent epidemiological studies of multiple sclerosis (MS) in the Russian Federation by major regions (standardized values in square brackets).

Region, Republic	Years	Prevalence per 100,000 Population (Standardized)	Incidence per 100,000 Population (Standardized)	F/M Ratio	Authors
European Russia					
Leningrad Region	1990–1994	28.8	2.2	–	Zaslavskyi, 2001 [9]
Smolensk Region	2006–2008 2005–2010	47.4 55.4	– 4.5	1.6 1.6	Maslova & Pysina, 2008 [10] Pysina, 2009 [11]
Republic of Karelia	2013–2018	61.2	2.7	–	Sirenev, 2019 [12]
Nizhny Novgorod Region Nizhny Novgorod City	1990–1992 2000–2002	36.0 38.0	– 1.6	– 1.7	Melnikova, 1992 [13] Kim, 2004 [15]
Ryazan Region	1990–1994	61.0	–	–	Leonov, 1995 [14]
Kaluga Region	2009–2012	54.7		2.03	Maslova, 2014 [16]
Yaroslavl Region	1994–2000 2010–2015 2015–2019	42.6 56.2 78.5	– 1.58 3.28	– – –	Spirin, 2003 [17] Kachura, 2008 [19] Spirin, 2020 [20]
Moscow Region	2001–2005	31.8 28.7	2.1 2.6	2.0 1.9	Lizhdvoy, 2006 [21] Sergeev, 2009 [22]; Kotov, 2012 [23]
Moscow City Central Part North-West	1989–1993 2008–2012	44.8 55.6 [48.2]	3.39 2.16 [1.88]	– 2.6	Gusev, 2002 [3] Boiko 2013, 2014 [24,25]
Bryansk Region	2008 2012–2016	40.6 48.1	– 1.9	– 3.0	Khudyakova & Gribova, 2009 [26] Yurchenko, 2016 [27]
Orel City	1995–2000	40.4	1.45	–	Khoroshilova, 2005 [28]
Kursk Region Kursk City	2015 2011–2015	78.4 70.8	– 3.75	– –	Laskov, 2017 [29] Gridnev, 2017 [30]
Chuvashia Republic	1984–1990 1998–2002 2002	19.0 31.0 37.6	– 1.6 2.57	– – –	Kuzmin & Egorova, 1995 [31] Sharov, 2004 [32] Egorova, 2004 [33]
Tatarstan Republic Kazan City	1999 2000 2010 2010	32.0 34.0 36.7 34.9	3.3 5.0 5.5 –	– – – 2.4	Khabirov, 2013 [34]
Bashkortostan Republic Ufa City	1999–2006 2018 2010–2016	35.3 [31.9] 47.9 46.5	3.5 3.0 –	– – –	Bakhtiyarova & Magzhanov, 2006 [35] Bakhtiyarova, 2019 [37] Bakhtiyarova & Goncharova, 2014 [36]
Stavropol Territory	1986–1991	23.8	–	–	Shevchenko, 1995 [38]
Karachay-Cherkessia Republic	1981–1991	16.9	–	–	Shevchenko, 1995 [38]
Rostov Region Rostov-on-Don City	2002–2006 2002–2006 2016–2018	24.6 26.3 55.0	1.7 1.7 1.6	– – –	Melnikova, 2008 [39], Goncharova, 2010 [40], Goncharova, 2019 [43]
Volgograd Region	1996–2000	31.9	9.8	–	Dokuchaeva, 2006 [44]
Dagestan Republic	2000–2004	5.7	0.2	1.2	Aysaeva, 2010 [47]
Ingushetia Republic Nazran City	2010–2015 2010–2015	13.2 31.2	1.76 –	– –	Goncharova & Uzhachov, 2018 [51]
Kabardino–Balkaria Republic, Nalchik	2010–2006 2010 2012–2017	13.0 34.3 –	1.76 – 1.9	– – 3.0	Zikhova, 2013 [49] Zikhova, 2019 [50]
Chelyabinsk City	2005–2009	50.7	–	–	Nikolaeva, 2010 [54]
Perm Territory	2007–2011	35.1	4.1	–	Zhelnin, 2013 [53]
Asian Russia					
Novosibirsk City	1994–1999 1999–2003	29.2 54.4	1.59 2.37	– –	Malkova, 2005 [55]
Tyumen Region Tyumen City	2001–2006 2014–2017 2001–2006	24.8 43.0 33.6	2.5 4.0 –	– 2.4 –	Sivertseva, 2006, 2009 [56,57,59] Sivertseva, 2010, 2017, 2018 [58,59,60]
Khanty-Mansi Autonomous Okrug	2015–2018	45.9	3.22	1.96	Sokolova & Dydymov, 2018 [61]
Tomsk Region	2000–2004 2006–2010	27.1 [24.0] –	– 2.9	1.5 2.0	Titova, 2004 [62] Alifirova, 2017 [63]
Altay Territory	2010 2018	41.2 56.3	1.1 2.6	– 2.0	Smagina, 2010 [64] Smagina, 2019 [65]
Iirlutsk City	2001–2005	26.5	–	1.85	Skliarenko, 2008 [66]
Sakha-Yakutia Republic Yakutsk City	2001–2006 2008–2016 2016–2018 2016	22.5 [21.2] 30.3 34.2 43.7	2.5 [2.2] 1.6 – –	2.7 – – –	Minurova, 2008 [69] Popova, 2017 [70] Nikolaeva, 2019 [71] Popova, 2017 [70]
Amur Region Blagoveshensk	1985–1998 2005–2010	54.3 31.4	3.87 1.54	– –	Karnaukh, 2009 [73] Karnaukh, 2019 [76]
Khabarovsk City	1985–1998 2018	40.5 30.1	2.90 3.48	– –	Karnaukh, 2011 [74] Karnaukh, 2019 [76]
Vladivostok City	2005–2010	–	2.50	–	Gavrilenko, 2012 [75]
Sakhalin Island	2005–2010 2018	– 30.1	3.95 3.48	1.35 –	Gavrilenko, 2012 [75] Karnaukh, 2019 [76]
Kamchatka Half-Island	2005–2010	–	2.60	–	Gavrilenko, 2012 [75]
Primorsky Territory	2018	33.0	4.15	–	Karnaukh, 2019 [76]

## References

[1] Browne P., Chandraratna D., Angood C., Tremlett H., Baker C., Taylor B.W., Thompson A.J. (2014). Atlas of multiple sclerosis 2013: A growing global problem with widespread inequity. Neurology.

[2] Boiko (Boyko) A., Deomina T., Gusev E., Favorova O., Sudomoina M., Turetskaya R. (1995). Epidemiology of multiple sclerosis in Russia and other countries of the former Soviet Union: Investigations of environmental and genetic factors. Acta Neurol. Scand..

[3] Gusev E.I., Zavalishin I.A., Boiko (Boyko) A., Khoroshilova N.L., Yakovlev A.P. (2002). Epidemiological characteristics of multiple sclerosis in Russia. Zh Nevrol Psikhiatr Im S. S. Korsakova.

[4] Boyko A., Smirnova N., Petrov S., Gusev E. (2016). Epidemiology of MS in Russia, a historical review. Mult. Scler. Demyelinating Dis..

[5] Poser C.M., Paty D.W., Scheinberg L., McDonald W.I., Davis A., Ebers G.C., Johnson K.P., Sibley W.A., Silberberg D.H., Tourtellotte W.W. (1983). New diagnostic criteria for multiple sclerosis: Guidellines for research protocols. Ann. Neurol..

[6] McDonald W.I., Compston A., Edan G., Goodkin D., Hartung H.P., Lublin F.D., McFarland H.F., Paty D.W., Polman C.H., Reingold S.C. (2001). Recommended diagnostic criteria for multiple sclerosis: Guidelines from the International Panel on the diagnosis of multiple sclerosis. Ann. Neurol..

[7] Belova A.N., Shalenkov I.V., Shakurova D.N., Boyko A.N. (2014). Revised McDonald criteria for multiple sclerosis diagnostics in Central Russia: Sensitivity and specificity. Mult. Scler. J..

[8] Odinak M.M., Bisaga G.N., Kalinina N.M., Akimov S.B., Semilutskaia I.B. (2000). Multiple sclerosis in Northern–West Region of Russia: Results of HLA–typing. Zh Nevrol Psikhiatr Im S. S. Korsakova..

[9] Zaslavsky L.G., Skoromets A.A. Dynamics of multiple sclerosis incidence in the Leningrad Region. Proceedings of the Abstracts of the 8th all–Russian Congress of Neurologists.

[10] Maslova N.N., Pisina A.M. (2008). Epidemiology of multiple sclerosis in the Smolensk Region. Bull. Siberskaya Med..

[11] Pisina A.M. (2009). Clinical and epidemiological features of multiple sclerosis in the Smolensk Region. Bull. Smolensk. Med. Acad..

[12] Sirenev I.M., Gerasimova–Maigal L.I., Sergeev A.M., Ivankva G.U., Orlov P.O. (2019). Epidemiology of multiple sclerosis in republic of Karelia at 2013–2018. Vestn. Yralskoy Med. Akad. Nayki.

[13] Melnikova T.V., Kabanovsky O.A., Yakubovich A.G. Multiple sclerosis in the Nizhny Novgorod Region. Proceedings of the Plenum of the Board of the Russian Society of Neurologists.

[14] Leonov G.A., Vasilevskaya L.V., Egorova M.V., Trifanova S. Multiple sclerosis in the Ryazan Region. Proceedings of the Abstracts of the VII All–Russian Congress of Neurologists.

[15] Kim E.R. (2004). Clinical Picture and Epidemiology of Multiple Sclerosis in the Nizhny Novgorod Region.

[16] Maslova N.N., Andreeva E.A., Belyi Y., Erochina E.A. (2014). Clinico–epidemiological and ophtalmological peruliarities of multiple sclerosis in the Kaluga Region. Ann. Nevrol..

[17] Spirin N.N., Kachura D.A., Kachura A.N., Boiko (Boyko) A.N. (2003). Influence of environmental factors on the incidence and prevalence of multiple sclerosis. Zh Nevrol Psikhiatr Im S S Korsakova.

[18] Kachura D.A. (2005). Clinical and Epidemiological Study of Multiple Sclerosis on the Model of the Urban Population of the Yaroslavl Region.

[19] Kachura D.A., Spirin N.N., Boyko A.N. (2008). Environmental aspects of multiple sclerosis. Cons. Med..

[20] Spirin N.N., Kasatkin D.S., Stepanov I.O., Shipova E.G., Baranova N.S., Vinogradova T.V., Molchanova S.S., Kisilev D.V., Shadrichev V.A., Spirina N.N. (2020). Dynamic of main epidemiological indexes of multiple sclerosis comparing data from registers pf patients in 1999 and 2019 (using as example Yaroslavl Region). Zh Nevrol Psikhiatr Im S S Korsakova.

[21] Iu. Lizhdvoi V., Sergeev S.A., Gurov A.N., Turovskii B.M. (2006). Epidemiology of multiple sclerosis in the Moscow region. Alm. Clin. Med..

[22] Sergeev S.A. (2009). Clinical and Epidemiological Characteristics of Multiple Sclerosis in the Moscow Region.

[23] Kotov S.V., Iakushina T.I., Iu. Lizhdvoi V. (2012). Clinical and epidemiological aspects of multiple sclerosis in the Moscow Region. Zh Nevrol Psikhiatr Im S. S. Korsakova..

[24] Boiko (Boyko) A.N., Kukel T.M., Lysenko M.A., Vdovichenko T.V., Gusev E.I. (2013). Clinical epidemiology of multiple sclerosis in the city of Moscow: Descriptive epidemiology on the example of a population of one of the city’s districts. Zh Nevrol Psikhiatr Im S. S. Korsakova.

[25] Boiko (Boyko) A.N., Kukel T.M., Lysenko M.A., Vdovichenko T.V., Gusev E.I. (2014). Clinical epidemiology of multiple sclerosis in Moscow. II. Modern clinical and demographic features on the example of the population of one of the districts of the city. Zh Nevrol Psikhiatr Im S. S. Korsakova.

[26] Khudyakova I.V., Gribova N.N. (2009). Clinical–epidemiological and bioradical aspects of multiple sclerosis in the city of Bryansk. Neiroimmunologia.

[27] Yurchenko Y.N., Yurchenko A.N., Smagina I.V. (2016). Epidemiology of multiple sclerosis in the Bryansk Region. Nevrologia, Nejropsihiatria, Psichosomatika (Neurology, Neuropsychiarty, Psuchosomatica).

[28] Khoroshilova N.L. (2005). Prevalence and Risk Factors of Multiple Sclerosis in the City of Orel.

[29] Laskov W.B., Logacheva E.A., Tretyakova E.E., Gridnev M.A. (2017). Clinical and epidemiological features of patients with multiple sclerosis in the Kursk Region. Nevrol. Nejropsihiatria Psichosomatika.

[30] Gridnev M.A., Logacheva E.A., Tretyakova E.E., Laskov W.B. (2017). Analysis of the availability of specialized medical care for patients with multiple sclerosis in the Kursk Region. Zh Nevrol Psikhiatr Im S. S. Korsakova..

[31] Kuzmin I.C., Egorova G.P. (1992). Clinical and epidemiological data on multiple sclerosis in the Chuvash Republic. Proceedings of the Plenum of the Board of the All–Russian Society of Neurologists.

[32] Sharov D.A. (2004). Clinico–Epidemiological Characteristics of Multiple Sclerosis in Chuvashia Republic.

[33] Egorova G.P. (2004). Dynamics of the Prevalence, Incidence, and Clinic of Multiple Sclerosis According to a Prospective Study.

[34] Khabirov F.A., Babicheva N.N., Esin R.G., Kochergina O.S., Garnet S., Khaibullin T.I. (2013). Clinical and socio–demographic characteristics of multiple sclerosis in the Republic of Tatarstan. Practicheskaya Medicina.

[35] Bakhtiyarova K.Z., Magzhanov R.V. (2006). Multiple sclerosis in ethnic groups of Bashkortostan Republic. Zh Nevrol Psikhiatr Im S. S. Korsakova..

[36] Bakhtiyarova K.Z., Goncharova Z.A. (2014). Multiple sclerosis in the Republic of Bashkortostan and the Rostov Region: Comparative epidemiological characteristics. Zh Nevrol Psikhiatr Im S.S. Korsakova..

[37] Bakhtiyarova K.Z., Galiullin T.R., Lutov O.V. (2019). The data of 10 years of experience of the regional register of multiple sclerosis. Zh Nevrol Psikhiatr Im S. S. Korsakova..

[38] Shevchenko P.P. Distribution and Clinical Characteristics of Multiple Sclerosis in the Stavropol Territory. Proceedings of the Abstracts of the VII All–Russian Congress of Neurologists.

[39] Melnikova A.V. (2008). Epidemiology, Clinical Features and Effectiveness of Treatment of Multiple Sclerosis in the Rostov Region.

[40] Goncharova Z.A. (2010). Clinical and epidemiological features of multiple sclerosis in the Rostov Region. Med. Bull. Yga Ross..

[41] Goncharova Z.A. (2013). Clinical and Epidemiological Characteristics of Multiple Sclerosis (a Prospective 20–Year Study).

[42] Goncharova Z.A., Pogrebnova Y.Y., Dolgyi B.A. (2018). Dynamics of epidemiological indicators of multiple sclerosis in the city of Rostov–on–Don. Zh Nevrol Psikhiatr Im S. S. Korsakova..

[43] Goncharova Z.A., Pogrebnov Y.U., Yarosh N.M. (2019). Is it still relevant to continue the epidemiological study of multiple sclerosis?. ] Zh Nevrol Psikhiatr Im S.S. Korsakova..

[44] Dokuchaeva N.N., Boiko (Boyko) A.N. (2006). Clinical and epidemiological study of multiple sclerosis in Volgograd city. Zh Nevrol Psikhiatr Im S. S. Korsakova.

[45] Dokuchaeva N.N. (2006). Clinical and Epidemiological Studies of Multiple sclerosis in Volgograd.

[46] Magomedov M.M., Khalitov I.A., Mikhailova B.I. (2009). Multiple sclerosis in Dagestan. Fundametalnyay Issled..

[47] Aysaeva Z.Z. (2010). Multiple Sclerosis in the Republic of Dagestan (Clinical and Epidemiological Study).

[48] Akhmadov T.K., Ismailov A.S., Chatayev G.S., Gamaev C.D. Epidemiology of multiple sclerosis in the Chechen Republic. Proceedings of the Abstracts of the X all–Russian Congress of neurologists.

[49] Zykova A.R., Beregova L.M., Tlapshokova L.B., Boiko (Boyko) A.N. (2013). Epidemiological characteristics of multiple sclerosis in the Kabardino–Balkaria Republic. Zh Nevrol Psikhiatr Im S. S. Korsakova.

[50] Zykova A.R., Tlapshokova L.B., Boyko A.N. (2019). The case of optionality with antibodies to aquaporin–4 in the Kabardino–Balkar Republic. Zh Nevrol Psikhiatr Im S. S. Korsakova.

[51] Goncharova Z.A., Uzhakhov R.M. (2017). Analysis of the prevalence and risk factors of multiple sclerosis in the Republic of Ingushetia. Zh Nevrol Psikhiatr Im S. S. Korsakova..

[52] Uzhakhov R.M., Goncharova Z.A. (2018). Epidemiological characteristics of multiple sclerosis in the Republic of Ingushetia. Zh Nevrol Psikhiatr Im S. S. Korsakova.

[53] Zhelnin A.V. (2013). Epidemiological and clinical features of multiple sclerosis in the Perm Region. Sarat. Zurnal Meditsinskich Issled..

[54] Nikolaeva L.I., Belskaya G.N., Lukashevich I.G., Kutepov N.V. (2010). Clinical and epidemiological aspects of multiple sclerosis in the southern Urals. Nevrol. Bull..

[55] Malkova N.A., Shperling L.P., Ryabukhina O.V., Merkulova E.A. (2006). Multiple sclerosis in Eastern Siberia a twenty–year prospective study in Novosibirsk. Zh Nevrol Psikhiatr Im S. S. Korsakova.

[56] Sivertseva S.A., Zhuravlev M.N., Murav’ev S.A., Boiko (Boyko) A.N. (2006). Epidemiology of multiple sclerosis in the Tiumen Region. Zh Nevrol Psikhiatr Im S S Korsakova..

[57] Sivertseva S.A. (2009). Epidemiological and Immunological Features of Multiple Sclerosis in the Tyumen Region.

[58] Sivertseva S.A., Kandala N.S., Zhuravlev M.N., Zakateĭ I.G., Boiko (Boyko) A.N. (2010). Multiple sclerosis in the native population of Yamal. Zh Nevrol Psikhiatr Im S S Korsakova.

[59] Sivertseva S.A., Zotov A.V., Kandal N.A., Baguhin D.V., Boiko (Boyko) A.N. (2017). Descriptive epidemiology of multiple sclerosis in the Tyumen Region. Zh Nevrol Psikhiatr Im S. S. Korsakova.

[60] Sivertseva S.A., Prilenskaya A.M., Kandala N.S., Vdovina N.E.I., Boyko A.N. (2018). Current state of multiple sclerosis epidemiology in the Tyumen Region] Zh Nevrol Psikhiatr Im S. S. Korsakova.

[61] Sokolova A.A., Dudumov O.N. (2018). Epidemiological features of multiple sclerosis in KHMAO–Yugra. Zh Nevrol Psikhiatr Im S. S. Korsakova.

[62] Titova M.A. (2004). Clinical and Epidemiological Characteristics of Multiple Sclerosis in the Tomsk Region.

[63] Alifirova V.M., Titova M.A., Gumenyuk Y.S., Semkina A.A. (2017). Dynamics of atypical forms of multiple sclerosis in the Tomsk Region for the period from 2012 to 2017. Zh Nevrol Psikhiatr Im S. S. Korsakova..

[64] Smagina I.V., Lichenko Y.N., Fedyanin A.S., Elchaninova S.A. (2010). Epidemiology of multiple sclerosis in the Altai territory. Nevrologicheskyi Zurnal.

[65] Smagina I.V., Elchaninova I.T., Elchaninova S.A. (2019). Multiple sclerosis in the Altai Region of Russia: A prospective epidemiological study. Zh Nevrol Psikhiatr Im S. S. Korsakova.

[66] Skliarenko O.V. (2004). Epidemiology and Clinical Characteristics of Multiple Sclerosis in Irkutsk.

[67] Nikolaeva T.Y., Popov V.S., Babenko L.I. (1995). Multiple sclerosis in Yakutia. Proceedings of the Abstracts of VII All–Russian Congress of Neurologists.

[68] Minurova A.R., Okoneshnikova L.T., Popova T.E., Kuzakova N.O., Boiko (Boyko) A.N. (2007). Clinical and epidemiological features of multiple sclerosis in Yakuts. Zh Nevrol Psikhiatr Im S. S. Korsakova.

[69] Minurova A.R. (2008). Clinical and Epidemiological Study of Multiple Sclerosis in Yakutia.

[70] Popova T., Nikolaeva T.N., Gorokhova N.U., Yilachova A.N., Okoneshnikova L.T. (2017). Multiple sclerosis in various ethnic groups of the Republic of Sakha (Yakutia): 10–year observation. Zh Nevrol Psikhiatr Im S.S. Korsakova.

[71] Nikolaeva T.N., Gorokhova N.U., Yilachova A.N., Okoneshnikova L.T. (2019). Prevalence of multiple sclerosis in Yakutia. Zh Nevrol Psikhiatr Im S. S. Korsakova..

[72] Karnaukh V.N., Barbash I.A. (2008). The course of multiple sclerosis in the Amur Region according to a prospective study for the period 1970–2007. Daln. Med. Zurnal.

[73] Karnaukh V.N. (2009). Dynamics of clinical presentations of multiple sclerosis in Amur Region for the period of 1960 to 2005. Zh Nevrol Psikhiatr Im S. S. Korsakova..

[74] Karnaukh V.N. (2011). Multiple Sclerosis in the Amur Region is a 35–Year Prospective Study (Epidemiology, Clinical Course, Outcomes).

[75] Gavrilenko A.A., Evdokimova Z.S., Vasikovskaia G.A., Boĭko A.N. (2012). Epidemiology of multiple sclerosis in the Primosky Krai and Far East regions. Zh Nevrol Psikhiatr Im S S Korsakova..

[76] Karnaukh V.N., Sherbanova N.E., Gavrilenko A.A., Glowinska N.G. (2019). Epidemiology of multiple sclerosis in the Far East. Zh Nevrol Psikhiatr Im S S Korsakova..

[77] Trojano M., Lucchese G., Graziano G., Taylor B.V., Jr (2012). Simpson, S.; Lepore, V.; Grand’maison, F.; Duquette, P.; Izquierdo, G.; Grammond, P.; et al. Study Group and the New Zealand MS Prevalence Study Group. Geographical variations in sex ratio trends over time in multiple sclerosis. PLoS ONE..

[78] Harbo H.F., Gold R., Tintoré M. (2013). Sex and gender issues in multiple sclerosis. Adv. Neurol. Disord..

[79] Alroughani R., Ahmed S.F., Behbahani R., Khan R., Thussu A., Alexander K.J., Ashkanani A., Nagarajan V., Al–Hashel J. (2014). Increasing prevalence and incidence rates of multiple sclerosis in Kuwait. Mult. Scler..

[80] Kearns P.K.A., Paton M., O’Neill M., Waters C., Colville S., McDonald J., Young I.J.B., Pugh D., O’Riordan J., Weller B. (2019). Regional variation in the incidence rate and sex ratio of multiple sclerosis in Scotland 2010–2017: Findings from the Scottish Multiple Sclerosis Register. J. Neurol..

[81] Valadkeviciene D., Kavaliunas A., Kizlaitiene R., Jocys M., Jatuzis D. (2019). Incidence rate and sex ratio in multiple sclerosis in Lithuania. Brain Behav..

[82] Wallin M.T., Culpepper W.J., Campbell J.D., Nelson L.M., Langer-Gould A., Marrie R.A., Cutter G.R., Kaye W.E., Wagner L., Tremlett H. (2019). US Multiple Sclerosis Prevalence Workgroup. The prevalence of MS in the United States: A population–based estimate using health claims data. Neurology.

[83] Onwuekwe I., Ezeala–Adikaibe B. (2011). Prevalence and distribution of neurological disease in a neurology clinic in enugu, Nigeria. Ann. Med. Health Sci. Res..

[84] Alla S., Pearson J., Debernard L., Miller D., Mason D. (2014). The increasing prevalence of multiple sclerosis in New Zealand. Neuroepidemiology.

[85] Dunn S.E., Gunde E., Lee H. (2015). Sex-based differences in multiple sclerosis (MS): Part II: Rising incidence of multiple sclerosis in women and the vulnerability of men to progression of this disease. Curr. Top. Behav. Neurosc..

[86] Baulina N., German G., Kiselev I., Popova E., Boyko A., Kulakova O., Favorova O. (2019). MiRNAs from DLK1–DIO3 imprinted locus at 14q32 are associated with multiple sclerosis: Gender–specific expression and regulation of receptor tyrosine kinases signaling. Cells.

[87] Baulina N., Kulakova O., Kiselev I., Osmak G., Popova E., Boyko A., Favorova O. (2018). Immune-related miRNA expression patterns in peripheral blood mononuclear cells differ in multiple sclerosis relapse and remission. J. Neuroimmunol..

[88] Matveeva O., Bogie J.F.J., Hendriks J.J.A., Linker R.A., Haghikia A., Kleinewietfeld M. (2018). Western lifestyle and immunopathology of multiple sclerosis. Ann. N Y Acad. Sci..

[89] Olsson T., Barcellos L.F., Alfredsson L. (2017). Interactions between genetic, lifestyle and environmental risk factors for multiple sclerosis. Nat. Rev. Neurol..

[90] Costenbader K.H., Gay S., Alarcón-Riquelme M.E., Iaccarino L., Doria A. (2012). Genes, epigenetic regulation and environmental factors: Which is the most relevant in developing autoimmune diseases?. Autoimmun. Rev..

